# Augmented feedback as a therapeutic approach for gait rehabilitation in patients with cerebral palsy: a systematic review

**DOI:** 10.3389/fresc.2026.1638091

**Published:** 2026-03-11

**Authors:** Leonie Hirsch, Natalie Mrachacz-Kersting

**Affiliations:** 1 Department of Neuroscience, Institute of Sport and Sports Science, Albert-Ludwigs Universität, Freiburg, Germany; 2 BrainLinks-BrainTools Center, IMBIT, Albert-Ludwigs University of Freiburg, Freiburg, Germany

**Keywords:** cerebral palsy, locomotion, gait rehabilitation, augmented feedback, motor learning, pediatric motor disability

## Abstract

**Objectives:**

To evaluate the effectiveness of augmented feedback (AF) in improving gait function in individuals with cerebral palsy (CP) and assess the strength of evidence across different gait parameters.

**Eligibility criteria:**

We included peer-reviewed interventional studies involving children or adults with CP who received AF during gait training, with gait-related outcomes assessed.

**Information sources:**

A systematic search was conducted in July 2025 across PubMed, Cochrane Library, IEEE Xplore, and PEDro database.

**Risk of bias:**

Risk of bias was assessed using the Cochrane Risk of Bias tool for RCTs and MINORS criteria for other study designs.

**Included studies:**

Of 477 screened records, 25 studies met inclusion criteria, comprising 612 total participants (409 intervention, 203 control). Studies included 13 single-session and 12 multi-session interventions.

**Synthesis of results:**

Using systematic evidence synthesis, velocity improvements showed strong evidencial support for AF, while ankle kinematics demonstrated moderate to strong evidence. Visual feedback had the most consistent effects across parameters, particularly for kinematic outcomes. Most other gait parameters (step length, stride length, cadence) showed inconclusive evidence due to conflicting findings across studies of varying quality.

**Limitations of evidence:**

High heterogeneity in protocols, outcome measures, and study quality (32% high, 52% moderate, 16% low quality) prevented a meta-analysis. Limited long-term follow-up data (only 5 studies) restricts conclusions about sustained effects.

**Interpretation:**

AF shows promise for enhancing gait velocity and ankle function in CP, particularly for spastic subtypes. However, evidence remains insufficient for widespread clinical adoption. Standardized protocols, larger sample sizes, and long-term follow-ups are essential for evidence-based implementation.

## Introduction

Cerebral palsy (CP) is a non-progressive neurological disorder caused by brain damage in the developing fetal or infant brain, affecting movement and posture (58). Motor impairments, particularly gait disturbances, are central features of CP, often accompanied by sensory, perceptual, cognitive, communication, and behavioral symptoms (59). CP is categorized into three subtypes—spastic, dyskinetic, and ataxic—based on the nature and extent of the brain lesion (1). These subtypes are further classified according to neurological involvement into unilateral (hemiplegia) and bilateral (diplegia or quadriplegia) presentations. Functional ability is assessed using the Gross Motor Function Classification System (GMFCS), which ranks motor abilities on a scale from levels I to V, with higher levels indicating more severe motor impairment (60).

As one of the leading causes of pediatric walking impairments, CP significantly limits mobility, restricting participation in daily activities and social engagement (61). Enhancing motor function through rehabilitation is crucial for improving the overall quality of life (2, 3). Despite advances in treatment, many individuals with CP face considerable challenges with mobility: 43.7% are not able to walk independently, where 11.1% require assistive devices, and 32.6% have minimal or no walking ability (62). There is a pressing need for novel rehabilitation approaches that target these specific motor deficits (63).

Augmented Feedback (AF), a technique that enhances the body's internal sensory system by providing external feedback to aid individuals in adjusting their movements (Moinuddin et al. 2021; Wälchli et al. 2016) has emerged as a promising tool for improving motor function and facilitating motor learning, particularly in rehabilitation settings (4). By providing real-time information about motor tasks, AF can enhance patient engagement and compliance during rehabilitation sessions (Merians et al. 2002). AF can be delivered through visual, auditory, or tactile stimuli, and is typically divided into two types: knowledge of results (KR), which provides feedback on the outcome of a movement, and knowledge of performance (KP), which focuses on the quality of the movement itself (5). Key factors in effective AF design include the specificity, timing, frequency, and level of guidance provided during the intervention.

Accumulating evidence suggests that external cueing exercises provided by AF may promote neuroplasticity, aiding motor recovery in patients with CP (Kleim and Jones 2008). Previous systematic reviews have explored various aspects of feedback and biofeedback in motor rehabilitation, including applications in CP. However, these were focused on upper limb motor skill learning (5), broad movement categories [Schoenmaker et al. 2022 (6);] or mixed neurological populations (7) limiting their relevance to gait-specific interventions in CP. While some reviews provide valuable theoretical frameworks or insights into specific feedback modalities, they often target other populations (8) or rely on studies involving healthy subjects and simple motor tasks (4). To date, no systematic review has focused exclusively on comparing different feedback modalities in gait rehabilitation for individuals with CP. This review addresses that critical gap by providing the targeted synthesis of evidence of AF for gait training in CP, allowing for a direct comparison of visual, auditory, and tactile feedback modalities within this specific clinical population.

Understanding how AF can influence the human gait cycle—particularly in the context of CP—presents a critical area for investigation. The human gait cycle consists of two main phases: the stance phase, when the foot is in contact with the ground, and the swing phase, when the foot moves forward. The stance phase comprises about 62% of the gait cycle and includes five sub-phases: initial contact, loading response, mid-stance, push-off, and pre-swing (1, 9). Gait impairments in CP vary depending on the clinical subtype. For instance, spastic hemiplegia often involves equinus, characterized by limited ankle dorsiflexion due to overactive plantar flexors or altered tissue properties (3, 10). Spastic diplegia is commonly associated with flexed knee gait, knee stiffness, in-toeing, and equinus, resulting from co-activation of antagonistic muscle groups (63, 68). Dyskinetic CP, on the other hand, is characterized by involuntary movements that disrupt normal gait patterns, while ataxic CP involves a wide-based gait and exaggerated arm swings to compensate for balance deficits (1).

Across all CP subtypes, individuals tend to exhibit simplified motor control strategies, using fewer muscle synergies—particularly at higher GMFCS levels. These limitations can significantly impact mobility and daily functioning (63, 69). These gait abnormalities underscore the urgent need for effective rehabilitation interventions that can restore or improve walking abilities (70, 71).

This systematic review aims to evaluate the effectiveness of different augmented feedback modalities, including visual, auditory, and tactile, for gait rehabilitation in individuals with CP. By focusing exclusively on this population and motor task, the review provides a focused synthesis of outcomes, informs future research and practice, and addresses a key gap in the existing literature.

## Materials and methods

The PRISMA guidelines (11) were strictly followed and the PRISMA checklist is provided in the Supplementary Material. A comprehensive literature search was conducted across multiple databases, including Cochrane Library, PubMed, IEEE Xplore and PEDro Library, to identify relevant literature. The search was completed as of July 11, 2025. Both manual and electronic searches were performed to ensure a thorough inclusion of relevant studies. The full search strings for Cochrane Library, IEEE Xplore and PEDro Library are available in the Supplementary Material. The search term used in the PubMed search was as follows:

(cerebral palsy OR CP OR “Cerebral Palsy"[Mesh]) AND (((augmented OR extrinsic OR external OR verbal OR visual OR video OR auditory OR haptic OR tactile OR sensory OR robot* OR multi-modal OR multimodal OR bio OR neuro OR vibrotactile) AND feedback) OR neurofeedback OR biofeedback OR visual augmented feedback OR audio augmented feedback OR multi-modal augmented feedback OR knowledge of performance OR knowledge of result* OR enhanced feedback OR feedback strateg* OR “Feedback, Sensory"[Mesh] OR “Feedback, Physiological"[Mesh] OR “Feedback"[Mesh] OR “Neurofeedback"[Mesh]) AND (walk* speed OR gait speed OR gait velocity OR walk velocity OR Step length OR Gait width OR mobility test OR spatiotemporal OR walk* test OR up and go OR Berg Balance Scale OR Physiological cost index OR Walking handicap scale OR (Functional Ambulation AND (Index OR Score)) OR step frequency OR cadence OR “Walking Speed"[Mesh] OR “Walk Test"[Mesh] OR “Gait Analysis"[Mesh] OR “Gait"[Mesh]).

**Figure 1:**
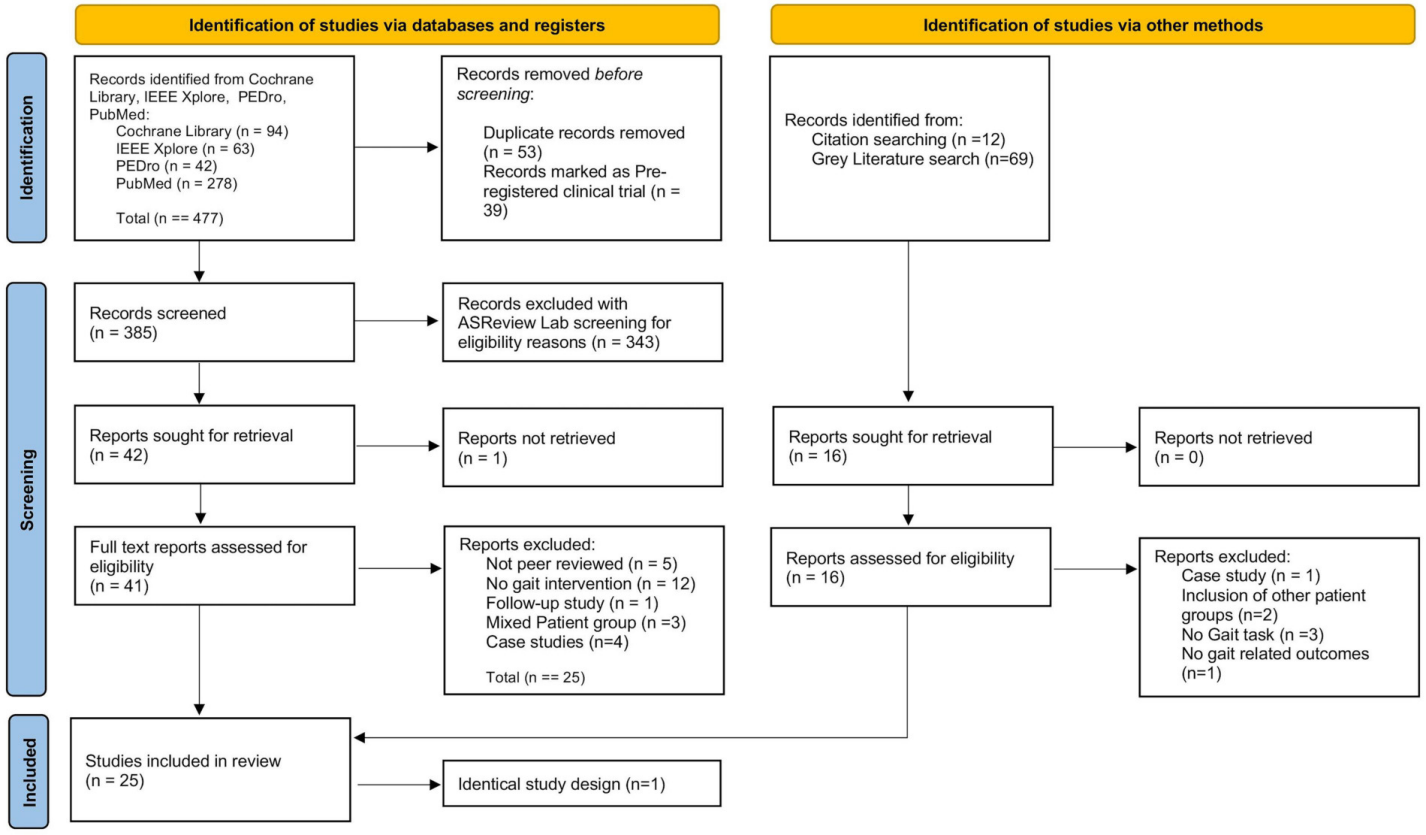
PRISMA flowchart of the conducted search.

A total of 477 results were obtained. After removing duplicates (*n* = 53) and pre-registered clinical trials (*n* = 39), 385 studies were eligible for screening. Additionally google scholar was searched for grey literature and reference lists of relevant articles were checked to ensure the identification of relevant articles that had been missed by the electronic search. In this manually performed search twelve articles were additionally identified. The metrics are shown in the PRISMA-Flowchart in Figure 1.

### Study selection

The study selection was performed with the Software ASReview Lab, a machine learning-based software for systematic reviews (12). After uploading metadata (titles and abstracts), an initial manual screening of 10% of the studies (*n* = 39) was conducted to train the software's AI algorithm. Two researchers independently performed this initial screening to ensure reliability in the training set. The remaining 385 studies were screened using this trained algorithm. Of the 346 papers reviewed, 42 were labeled as relevant for further analysis.

The use of ASReview allowed for a more efficient screening process while maintaining transparency and traceability of inclusion decisions (12). However, machine learning-based screening relies heavily on the quality of the initial training data and may risk overlooking relevant studies if the training set is not representative. To avoid this, independent screening was used for the initial training, and the final selection was verified by two researchers to ensure consistency.

Studies were included if they (1) involved patients diagnosed with cerebral palsy (CP) or comparisons between CP patients and healthy controls, (2) the intervention involved a task requiring participants to walk during the intervention and assessed the effects of augmented feedback on gait rehabilitation, (3) a comparisons was made between CP patients and healthy controls, different rehabilitation methods, or through pre- and post-intervention assessments within the same group, (4) the outcomes included quantitative measures related to gait performance, such as spatiotemporal, kinematic, kinetic, and electromyographic parameters, (5) the original research was original and peer-reviewed, was (6) conducted on humans, and lastly (7) the articles had to be published in English or German.

Studies were excluded if they (1) involved etiologies other than CP, (2) used interventions based on mirror therapy, electrical stimulation, or exoskeletons (unless the feedback was analyzed separately) and (3) they were in the format of commentaries, expert reports, pre-registered trials, reviews, meta-analyses, and non-peer-reviewed publications.

Studies involving robotic or electromechanical exoskeletons were excluded to isolate the effects of augmented sensory feedback (e.g., auditory, visual, or tactile) delivered without mechanical assistance. The aim was to focus on feedback-driven motor learning rather than assistive gait devices.

Given that only a few high-quality randomized controlled trials were available on this topic on augmented feedback for gait rehabilitation in cerebral palsy, this review intentionally included a range of study designs. All included studies assessed gait parameters, enabling a degree of comparability despite methodological differences. With this procedure the study aimed to provide a broad overview of current evidence, including both experimental and preliminary studies. While study designs vary, the goal is to offer an informative synthesis to inform future research and clinical practice. Due to the heterogeneity in study designs, intervention protocols, and outcome measures, a quantitative meta-analysis was not feasible. Instead, a qualitative synthesis was conducted to summarize the levels of evidence, identify trends, and highlight gaps in the literature.

Full-text screening was conducted on 41 studies. Studies were excluded if they were: Non-peer-reviewed publications (*n* = 5), no gait task in the intervention (*n* = 12), inclusion of other patient groups (*n* = 3), case studies (*n* = 4) and focus on long-term follow-up only (*n* = 1). Fourteen studies met the inclusion criteria. Two papers with similar study designs but different outcome measures were also included (13, 14). A manual literature search identified 12 potentially relevant studies, five of those were excluded, leaving 7 studies via citation searching. In total, 25 studies were included in this systematic review.

### Data extraction and study quality

Data from the studies were extracted using a pre-developed electronic data sheet. The extracted information included study design, participant demographics (e.g., sex, age, weight, CP type, GMFCS level), feedback protocol characteristics, measurement tools, and reported outcomes. Gait performance measures, such as spatiotemporal, kinematic, and kinetic parameters, were extracted. One reviewer performed the data extraction, which was then reviewed and verified by a second researcher to ensure completeness and accuracy. Additionally, data for assessing the quality of the studies was first assessed by one reviewer and subsequently discussed with a second reviewer to reach agreement for each study.

The methodological quality of non-randomized studies was assessed using the MINORS tool (15). This tool evaluates 12 criteria, with a maximum score of 16 for non-comparative studies and 24 for comparative studies. Higher scores indicate stronger methodological quality. For randomized controlled trials (RCTs), the Revised Cochrane Risk-of-Bias Tool 2 (RoB 2) was used (16). RoB 2 assesses bias in randomization, adherence to interventions, outcome measurement, and data reporting. Studies were categorized as low, unclear, or high risk of bias, with results presented in a risk-of-bias figure. Both tools provided insights into the strengths and limitations of the studies, contributing to a comprehensive analysis of the reliability of the findings in this systematic review.

### Level of evidence synthesis

Building upon the heterogeneity of study designs and outcomes outlined in Section 2.2, a quantitative meta-analysis was not feasible. Instead, a level of evidence synthesis was performed to assess the consistency and reliability of findings across studies. For each outcome parameter, the direction of effect reported in the included studies was examined. When 75% or more of the studies analyzing a specific parameter demonstrated effects in the same direction, the findings were classified as consistent. The overall level of evidence was determined based on both the consistency of findings and the methodological quality of the contributing studies, using the following criteria: evidence was categorized as strong when consistent findings (≥75%) were observed in two or more high-quality studies. Evidence was considered moderate when consistent findings were present in at least 67% of high-quality studies. Limited evidence was assigned when consistent effects were found in a single high-quality study or in one or more low- or moderate-quality studies. Inconclusive evidence was assigned when results were inconsistent (i.e., pointed in different directions), regardless of study quality. In cases where multiple high-quality studies were available, only those high-quality studies were considered in determining the level of evidence.

## Results

A total of 25 out of 397 studies met the inclusion criteria. The results of these studies are presented in the following sections.

### Participants

A total of 612 participants were included across all studies, with 409 participating in intervention groups and 203 in control groups. Within the control groups, 121 participants were typically developing (TD). Eleven studies did not include a control group (13, 17–25, 72). Participants' ages ranged from 3 to 65 years, with a mean age of 11.56 ± 5.45years. The sex distribution in the study groups was 202 males to 143 females, though five studies did not report the sex of their participants. Eight studies included adults (19, 20, 22, 25–29). The majority of participants had spastic cerebral palsy (CP), with 352 cases, including spastic hemiplegia, diplegia, and quadriplegia. Most studies used GMFCS I-III as an inclusion criterion, with 71 participants classified as GMFCS I, 92 as GMFCS II, and 11 as GMFCS III. Eleven studies did not report GMFCS levels (17, 18, 21, 22, 27–33). More detailed information on participant demographics is provided in Tables 1 and 2.

**Table 1:** Extracted details of the included single-session studies.

Reference	Study design	N (ig)	M:f	Cp characteristics	Age	Control group	Intervention/gait task	Frequency+duration	Comparison/control condition	Outcome measure	Feedback provided	Significant outcome parameters	Study quality
(13)(1)	Repeated measures design	25	15:07	Spastic CP (hemiplegia, diplegia); GMFCS I: 10, II: 12	10.5 ± 3.1	/	Treadmill walking with visual feedback comparing knee extension and ankle power through bar graph or avatar	single session	VR scene without Feedback	Spatiotemporal paramters; kinematic parameters (joing angles, joint work)	KP (v), concurrent, Continous, Descriptive	step length ↑ step width ↓ ankle power generation at push-off ↑ knee RoM ↑ Max knee ext (stance) ↑ Max knee ext Initial Contact ↑ Hip RoM ↑ hip abduction (swing) ↑ Max hip ext ↑	Moderate
(34)	Cross-sectional crossover trial with a repeated measures design	25	11:14	Spastic CP (hemiplegia, diplegia, and quadriplegia); GMFCS I:15:; II:10	14.7 ± 1.6	/	30 m walking with visual feedback on walking speed through different cues from a headset	single session	walking without feedback (30 m)	Mean speed, percentage of time spent above or around target speed + time to reach target speed	KP (v), concurrent, Continous, Pescriptive	velocity ↑ time above target speed ↑ time to reach the target speed ↓	Low
(14) (2)	Repeated measures design	25	16:09	Spastic CP; GMFCS I: 10, II: 12	10.4 ± 3.1	27 TD	Treadmill walking with visual feedback comparing knee extension and ankle power through bar graph or avatar	single session	TD walking without feedback	Spatiotemporal paramters; kinematic parameters (joing angles, joint work)	KP (v), concurrent, Continous, Descriptive	number of synergies required to explain at least 90% of muscle activation ↑ change in measures of Total variance accounted for during trials →	Moderate
(35)	Within-subject comparative study (repeated measures design)	16	/	Spastic CP; GMFCS I-III	11.3 ± 3.4y	11 TD	Treadmill walking comparing visual feedback on knee extension and hip Extension	single session	TD walking without feedback	Spatiotemporal paramters; kinematic parameters (joing angles, gait profile score (GPS)	KP (v), concurrent, Continous, Descriptive	stride length ↑ swing time ↑ Dorsiflexion (late stance) ↑ Max knee ext ↑ Max hip ext ↑ Trunk + Pelvis RoM ↑	Moderate
(20)	Feasibility study with a randomized crossover design	12	09:03	CP; GMFCS I-III (I:5;II:5;III: 2)	18.91 ± 8.4y	/	Treadmill walking with: only visual feedback on plantar preasure in virtual enviroment, only walking with exsceleton; and the combination	single session	Incline walking without feedback/ assistance	ankle, knee, and hip mechanics + plantar flexor and knee extensor EMG activity	KP (v), concurrent, Continous, Pescriptive	peak ankle momentum ↑ mean positive ankle power (stance) ↑ max knee extension ↑ peak + intrgrated SOL EMG ↑ peak+intrgrated Gastro EMG ↑ peak+intrgrated VL EMG ↑	Moderate
(36)	observational cross-sectional feasibility study	18	10:08	Spastic CP (hemiplegia, diplegia); GMFCS I: 9, II: 9	10.5 ± 2.9	12 TD	Treadmill walking with visual feedback on SOL/GM EMG activity through controlling a monkey in virtual enviroment	single session	TD walking in gaming enviroment without Feedback	EMG activity; Joint angles	KP (v), concurrent, Continous, Descriptive	peak ankle power (push-off) ↑ max knee extension ↑ muscle activity Triceps Surae (early stance) ↓	Moderate
(24)	Single-group repeated measures design	13	06:07	Spastic CP (diaplegia); GMFCS I = 9; II = 4	6 ± 2.08	/	10 m walking with auditive feedback on Dynamic foot pressure index through sound of negative result	single session	walking without feedback/ instructions	dynamic foot pressure index	KP (a), concurrent, Continous, Descriptive	dynamic foot pressure index ↑	Moderate
(26)	Experimental study with multiple intervention arms and control groups	20	V: 03:07; A: 04:07	CP	V: 11.1 ± 6.24; A: 13.3 ± 5.9	V: 7; A; 8 TD	10 m walking with visual (v) and auditory (a) feedback on through step length (a) + motion of the patient (a) through dynamic traverse lines (v, VR) + clicking sound (a)	single session	pre-post (20 min); TD also underwent a/v feedback programm	Spatiotemporal paramters	KP (a vs. v), concurrent, Continous, Descriptive	stride length ↑ velocity ↑	Moderate
(19)	Validation study with a repeated measures crossover design	8	07:01	Spastic CP(hemiplegia; diplegia); GMFCS I:2; II:4; III:2	14.38 ± 2.34	/	treadmill walking with auditive (a) + visual (v) Feedback on SOL EMG activity and plantar presure through a bar graph (v) + sound signal (a)	single session	walking without feedback	Kninematic paramters (joint angles), EMG anctivity	KP (v)/ KR (a), concurrent, Continous, Pescriptive	mean SOL activation ↑ mean+peak Gastro activation ↑ Ankle co-contraction index ↑	High
(30)	cross sectional study	12	09:03	CP type N/A; GMFCS I &II	11.4 ± 3.6	age matched healthy controls (*n* = 12)	Participants walked a 10-meter path with a wearable sensor providing real-time tactile vibration feedback when trunk sway exceeded set limits, aiming to improve trunk stability and gait control.	single session	walking without tactile feedback within group; Age-matched typically developed children	lower extremity movement trajectories and ROM; Spatial-temporal parameters	KP (t), concurrent, continuous, descriptive	Stance phase (%) ↓: 73.91% → 63.53% Step width ↓: 0.20 m → 0.18 m Step time ↓: 1.55 s → 0.73 s Cadence ↑: 59.25 → 63.63 steps/min Walking speed ↑: 0.52 → 0.61 m/s Pelvic tilt ↓: 4.2° → 2.9°; obliquity ↓: 13.1° → 9.2°; rotation ↓: 17.5° → 11.2° Ankle plantarflexion at initial contact ↓ Ankle dorsiflexion (stance & swing) ↑ Ankle ROM ↑: 23.5° → 34.8° Knee ROM ↑: 31.7° → 36.7° Hip ROM ↑: 31.7° → 36.7°	High
(21)	a repeated-measures analysis	12	12:00	spastic quadriplegia; GMFCS I	13 ± 3.5	/	Participants walked along an 8-meter platform guided by rhythmic auditory stimulation delivered via loudspeakers, using 2-, 4-, and 6-beat rhythm patterns to improve gait timing and coordination.	single session	pre–post test design	Time and distance gait parameters including walking velocity, cadence, stride/step length, step width, step/stride time, single and double support (% and time), double step length, opposite foot lift (%), and limp index	KP (a), concurrent, continuous, prescriptive	velocity (6-beat↑, 4,2-beat↓) cadence ↓ (6,4,2-beat) step length ↓ (4,2-beat) Double support time ↑ (6,4-beat) opposite foot lift ↓ (6,4,2-beat) double-step length ↓ (4,2-beat) Double support (6-beat↓, 4,2-beat↑) Single support (6,4-beat↓, 2-beat↑) double support time ↑ (6-beat, 4-beat) stride length ↓ (2-beat) Limp index ↓ (6,4,2-beat) Step width ↑ (6,4-beat)	Moderate
(27)	a repeated-measures analysis	14	09:05	bilateral spasticity; GMFCS I -III	25.64 ± 7.31	helathy controls (*n* = 30)	Walking was performed over 10 meters with rhythmic auditory stimulation combining a metronome beat matched to individual cadence and live keyboard-played simple chords to promote immediate gait improvements.	single session	epeated-measures analysis with/ without RAS; Comparison healthy controls	Temporospatial parameters (cadence, velocity, stride/step length and time, single/double limb support, stance and swing phase, side-to-side asymmetry); kinematic angles of pelvis, hip, knee, ankle, and foot across sagittal, coronal, and transverse planes; Gait Deviation Index (GDI).	KP (a), concurrent, continuous, descriptive	pelvic anterior tilt ↓ hip flexion ↓ Gait Deviation Index (GDI) ↑ step length asymmetry ↓ (household ambulators)	High
(23)	cross-sectional design	10	07:03	hemiplegic CP; GMFCS I: 9, II: 1	4.98 ± 0.8	/	The intervention involved children walking across a GAITRite system walkway while observing a virtual reality screen that displayed modulated optic flow speed (slow, medium, fast). The virtual environment mimicked walking in a park.	single session	3 optic flow speed conditions: Slow (0.25× the normal speed) Normal speed Fast (2× the normal speed)	study assessed spatiotemporal gait parameters using a GAITRite system, including walking velocity, cadence, stride length, step length, normalized step length, single and double support time, and base of support.	KP (v), concurrent, Continous, Pescriptive	fast optic flow: walking velocity ↑ cadence ↑ normalized step length ↑ base of support ↑ single support cycle ↑ slow optic flow: walking velocity ↓ cadence ↓ normalized step length ↓ base of support ↓ single support cycle ↓	High

**Table 2:** Extracted details of the included multi-session studies.

Reference	Study design	n (IG)	M:F	CP characteristics	Age	Control group	Intervention/gait task	Frequency+Duration	Comparison/Control Condition	Outcome Measure	Feedback provided	significant outcome parameters	Study Quality
(33)	RCT	30	15:15	Spastic CP (hemiplegia); GMFCS I, II	6–10y	15 children with spastic CP	walking with auditory Feedback on cadence on steps through rythmic auditory beats	Intervention	pre-post (4 months);traditional rehabilitation programm	Spatiotemporal paramters	KP (a), concurrent, Continous, Perscriptive	step length asymmetry ↓ stride length asymmetry ↓ stance time asymmetry ↓ swing time asymmetry ↓	Low
(18)	Pre-post intervention study (single-group pre-post design)	8	/	Spastic/ mixed CP	6 ± 1.66y	/	walking with auditory Feedback on heel preasure through buzzing sound	Intervention (4 months)	pre-post (3 months-1y); walking without feedback	passive ankle dorsiflexion, number of seconds of heel contact	KP (a), concurrent, Continous, Descriptive	the total duration of heel down ↑ total number of heel strikes ↑ dorsiflexion with the knee extended (IC) ↑	Low
(32)	RCT	30	17:13	Spastic CP + E19(hemiplegia)	7.05 ± 0.78	15 children with spastic CP	different walking sytyles with auditory feedback on step length and walking speed through rythm + speed of music	Intervention (8 weeks, 5 sessions/ week)	pre-post (3months); Control group recieved traditional gait training programme	Spatiotemporal paramters	KP (a),concurrent, Continous, Descriptive	velocity ↑ stride length ↑ cadence ↓	Moderate
(37)	RCT	30	/	Spastic CP(diaplegia); GMFCS II	5.23 ± 0.52	15 children with spastic CP	waking with visual feedback on plantar preasure through visualization of seven pressure areas of the foot	Intervention (24 sessions)	pre-post (2 months); therapeutic exercise program for 1 h 30 min walking without feedback	Spatiotemporal paramters	KP (v), concurrent, Continous, Descriptive	step length ↑ step width ↓ cadence ↑ velocity ↑	Moderate
(17)	Two-period crossover design	7	04:03	Spastic CP (hemiplegia)	10.57 ± 2.56	/	walking with auditive (a) + visual (v) Feedback on Triceps surae EMG activity through a dynamic line (v) and sound signal (a)	Intervention (8 sessions)	pre-post; follow-up (1w); Physical therapy (2 groups, both did intervention+control condition)	Spatiotemporal paramters; kinematic parameters (joing angles, joint work)	KP + KR (v); KR (a), V:Concurrent+Terminal; A: Terminal After every step; Continous, v: Perscriptive; a: Descriptive: correct aspects of performence	Peak power generation at the ankle (push-off) ↑	Moderate
(28)	Randomized controlled trial repeated-measures analysis comparing pretreatment and posttreatment tests	15	10:05	Bilateral spasticity/GMFCS N/A	27.3 ± 2.4	neurodevelopmental treatment (NDT) (*n* = 13)	Participants received 30-minute gait training sessions, three times weekly, comparing rhythmic auditory stimulation with metronome and live keyboard cues versus traditional neurodevelopmental Bobath therapy for gait enhancement.	Intervention (9 sessions)	pre–post test design; Rhythmic Auditory Stimulation (RAS) group; Neurodevelopmental Treatment (NDT/Bobath) group	Temporal gait parameters (cadence, velocity, stride/step length and time, stance and swing phase); kinematic joint angles (pelvis, hip, knee, ankle, foot); Gait Deviation Index (GDI)	KP (a), concurrent, continuous, descriptive	cadence ↑ walking velocity ↑ stride length ↑ step length ↑ stride time ↓ step time ↓ pelvic anterior tilt ↓ hip flexion (minimal) ↓ Gait Deviation Index (GDI) ↑ swing phase ↑	High
(38)	pre-post treatment experimental desig; pilot study	11 (9 included in analysis)	04:05	spastic CP (Hemiplegia:4 Diplegia.3 Quadriplegia:2); GMFCS Levels: I: 2; II: 4; III: 3	6.89 ± 2.98	/	Children walked on a 14-meter walkway receiving rhythmic auditory stimulation via metronome or embedded music beats at normal and increased tempos, with rest intervals and pre/post cueing walks to measure carry-over effects.	Intervention (3 sessions)	walking without cueing; 2 different cueing sppeds (initial cadence/ initial cadence +5%)	Cadence (steps in 30 s×2); walking velocity (meters per minute); stride length calculated as (velocity/cadence) × 2.	KP (a), concurrent, continuous, prescriptive	velocity ↑ stride length ↑ cadence ↑ (with more sessions) cadence ↑ (with higher GMFCS level) cadence ↑ (with increased cueing speed) velocity ↑ (with higher GMFCS level) stride length ↑ (with higher GMFCS level)	Moderate
(22)	two-group, pre-post interventional design	13 (simple:6; complex:7)	4:9 (simple 2:4; complex: 2:5)	Diplegic CP	19.75 (simple: 20.0 ± 2.8 complex: 19.5 ± 5.0)	/	Using 30-minute sessions, participants walked while receiving rhythmic auditory stimulation with either simple or complex chord progressions played live by a music therapist, with tempo adjustments to optimize gait stability over 4 weeks.	Intervention (12 sessions)	pre-test and post-test design for both: RAS with simple chords/RAS with complex chords	Spatiotemporal parameters (cadence, velocity, step length, step time, step width, single/double support); kinematic parameters (dynamic joint ROM for pelvis, hip, knee, and ankle across multiple planes).	KP (a), concurrent, continuous, prescriptive	cadence ↑ walking velocity ↑ stride length ↑ hip extension at terminal stance ↑ ankle plantar flexion during preswing ↑ (complex RAS > simple RAS) ankle dorsiflexion ROM ↑ (complex RAS > simple RAS)	High

### Risk of bias

**Figure 2:**
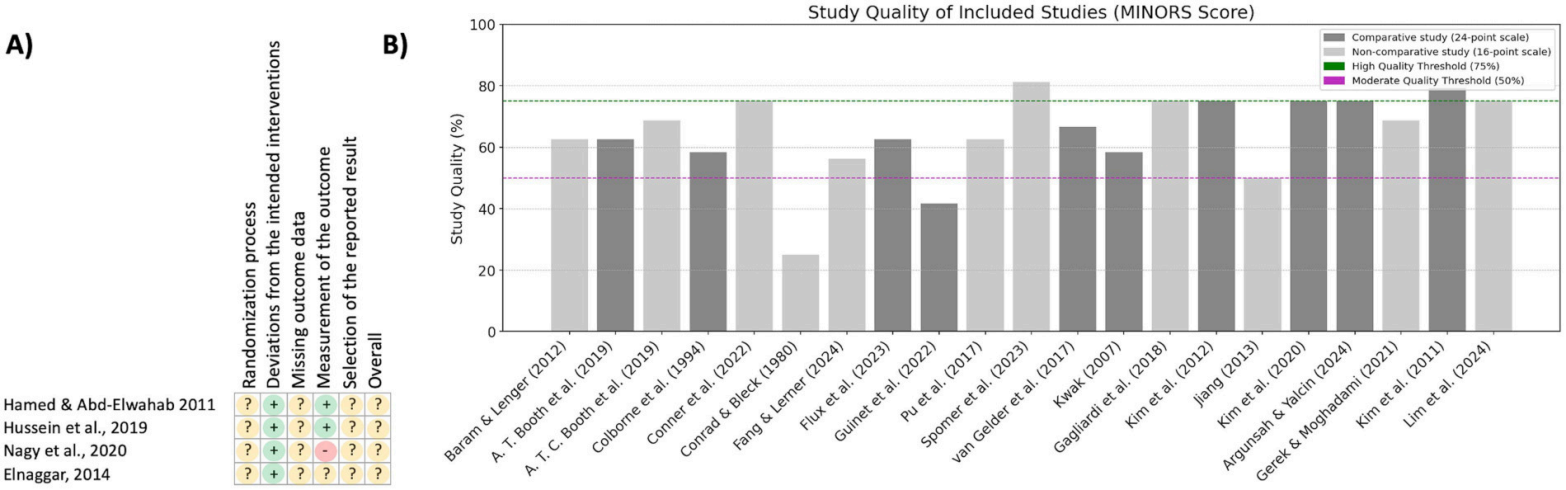
**(A)** quality assessment of randomized studies according to the RoB2 assessment, **(B)** quality assessment of non-randomized studies according to the MINORS assessment with the total value adjusted to percentage on the *x*-axis.

The results of the risk of bias analysis are presented in Figure 2A for the five RCTs studies and in Figure 2B for the remaining 16 studies. All of the included studies showed at least “some concerns” in their study designs. The results of the MINORS assessment were as follows:

T. Booth et al. (13) 15/24; A. T. C. Booth et al. (14) 11/16; Argunsah and Yalcin (30) 18/24; Baram and Lenger (26) 10/16; Colborne et al. (17) 14/24; Conner et al. (19) 12/16; Conrad and Bleck (18) 4/16; Fang and Lerner (20) 9/16; Flux et al. (36) 15/24; Gagliardi et al. (39) 12/16; Gerek and Moghadami (21) 11/16; Guinet et al. (34) 10/24; Jiang (38) 8/16; Kim et al. (27) 19/24; Kim et al. (28) 18/24; Kim et al. (22) 18/24; Kwak (29) 14/24; Lim et al. (23) 12/16; Pu et al. (24) 10/16; Spomer et al. (25) 13/16; van Gelder et al. (35) 16/24.

### Characteristics of the included studies

The studies differed in the duration of the intervention with 13 studies were conducted as a single session intervention (13, 14, 19–21, 23, 24, 26, 27, 30, 34–36), and 12 studies across multiple sessions (17, 18, 22, 25, 28, 29, 31–33, 37–39). The following sections will present and compare these separately. Table 1 summarize the characteristics of the single-session studies, while Table 2 and 5 represent the multi-session studies.

**Figure 3:**
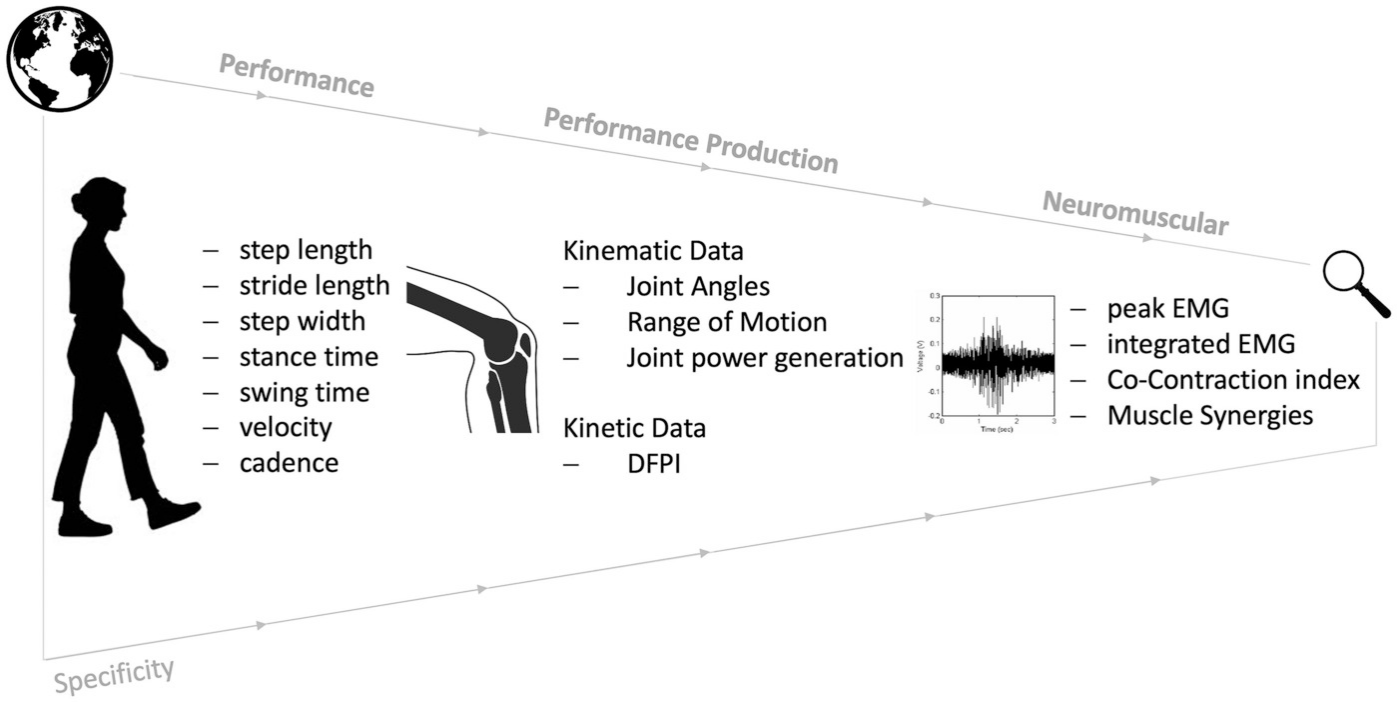
Categorization of the outcome parameters.

Outcome measures were grouped into three categories as shown in Figure 3: performance measures including basic gait parameters, performance production measures covering biomechanical and kinetic measures, and neuromuscular parameters. In the following section, the study results will be presented according to this structure.

### Enhanced performance parameters

Step length improvements were reported in three single session studies (13, 23, 30), whereas Argunsah and Yalcin (30) did not report if these were indeed significant One study found a significant decrease for step length for slower rhythms of RAS (rhythmic auditory stimulation) (21) which may have been induced through reduced visual flow (compared to normal velocity) as found by Lim et al. (23). RAS may also lead to significantly reduced step length asymmetry for CP patients with slower functional gait (27). An increased stride length was reported in two studies (26, 35). Gerek & Moughadami (21) also reported a reduced stride length in the slowest rhythm of the RAS. Two studies additionally quantified compensatory increases in step width with increasing walking speed (13, 21). In the multi-session studies significant step length increases were found in four studies (22, 28, 31, 33, 39). Stride length improved in five studies (22, 28, 32, 38, 39) while others showed no significant changes in step length (21, 26, 30, 34) or stride length (25, 31).

Visual, auditory, RAS and tactile feedback improved walking speed after only one intervention session (21, 23, 26, 30, 34). Visual, auditory and RAS also increased walking velocity in studies conducted over multiple sessions (22, 28, 29, 31, 32, 38, 39). However, in their findings Kwak (29) report that the walking speed only increased for the group receiving Therapist guided training (compared to Self guided training) and Jiang (38) found that level of gross motor functioning had a statistically significant main effect on velocity. The findings of changes in step frequencies showed heterogenous results with some studies providing evidence for an increased step frequency for a single session (23, 30) and for multiple sessions (22, 28, 37, 38) while others report decreases (23, 32). This, however, may also be due to the design of the gait task given, since in some studies the goal was to increase walking speed (26, 30–32, 34, 37, 39), whereas in others the walking speed was set to a fixed pace (13, 19–22, 25, 27–29, 35, 36, 38). Lim et al. (23) demonstrated that altering optic flow (defined as the pattern of visual motion perceived on the retina relative to walking speed) can modulate step frequency, increasing it with faster optic flow and decreasing it with slower optic flow.

Changes in stance and swing time during gait were reported in only a few studies. In the single-session study by van Gelder et al. (35), an increased swing time on the affected leg was observed, suggesting improved gait symmetry. Similarly, Nagy et al. (33) found improvements in stance and swing phase symmetry following multiple sessions of auditory feedback intervention. In another single-session study, Argunsah and Yalcin (30) reported a decrease in pathologically extended stance percentage using tactile biofeedback. Among multi-session studies, Gagliardi et al. (39) also observed a decrease of stance time, while Kim et al. (28) noted modest normalization of the stance and swing phases with RAS. Additionally, Kim et al. (22) reported a significant increase in swing phase duration and a significant reduction in stance time compared to neurodevelopmental training therapy.

### 3.5 Performance production measures

The majority of the studies focused on improving joint function. Most studies analyzed ankle, knee, and hip joints individually, with kinematic data analyzed more frequently than kinetic data.

### Ankle kinematics

In single-session studies, positive effects on ankle kinematics were reported, with increased mean and peak ankle power generation during push-off (13, 20), and improved ankle momentum during the stance phase (20). The increase occurred in studies that provided visual feedback. In Fang and Lerner's (20) feedback protocol, participants were required to walk on an incline. This induced greater ankle power generation and therefore increased dorsiflexion at heel strike. One study noted a significant increase in ankle dorsiflexion during late stance after visual feedback on hip kinematics but not after feedback on knee kinematics (35). This increase in ankle dorsiflexion during both stance and swing phases as well as a reduction in excessive ankle plantarflexion at initial contact was shown by Argunsah and Yalcin (30) using tactile feedback, resulting in improved ankle ROM. In contrast two studies using RAS and one utilizing combined auditory and visual feedback did not observe significant differences in ankle kinematics or range of motion (RoM) (19, 21, 27).

In multi-session studies, improvements in dorsiflexion during initial contact was one reported outcome measure (18), particularly benefiting patients with equinus gait. Audiovisual feedback on muscle activation enhanced plantarflexion during push-off (17, 25) and the peak-power generation in the ankle (17). Gagliardi et al. (39) using visual feedback demonstrated significant improvements in ankle ROM for flexion-extension during both stance and swing phases, along with increased ankle peak power. Kim et al. (28) found no statistical differences in ankle kinematic parameters with RAS, while Kim et al. (22) reported significantly greater ankle plantar flexion at push-off and increased dorsiflexion ROM in the complex RAS group compared to the simple RAS group (i.e., either a simple or complex chord progression was played as RAS).

### Knee kinematics

Knee kinematics were the second most analyzed joint given the gait deviations of flexed knee joint gait and stiffness in CP. Single-session studies reported significant increases in peak knee extension (13, 20, 35, 36). Two studies however found no significant changes in the knee joint kinematics (19, 27). Greater improvements were observed when feedback was provided on joint angles (13, 35) compared to feedback focused on muscle activation or plantar pressure (20, 36). Argunsah and Yalcin (30) reported lower knee flexion during both “with” and “without” tactile feedback conditions, with increased knee ROM.

Multi-session studies also highlighted positive effects, such as increased knee flexion during the swing phase (25), reduction of excessive knee flexion at initial contact (39), increases in knee extension during a passive RoM test (18) and a decrease in knee joint displacement during mid-stance (31). Three studies, also reported no significant changes in knee kinematics (17, 22, 28), however one of those were comprised of a small sample size (17). In a two week follow up measurement the knee flexion was still increased (25) in the AF group.

### Hip kinematics

As excessive hip flexion during the stance phase is another common feature of CP gait, particularly in the crouch gait, the hip joint was another joint to target for AF interventions. Single-session studies found increased hip RoM (13, 27, 35), but also reported some compensatory movements, such as increased hip abduction, during the swing phase (13). Other studies found no significant changes in hip kinematics (19, 20). As for the studies quantifying changes across the knee joint, studies with significant changes across the hip joint used feedback on joint kinematics, while those with non-significant results gave feedback with regards to changes in plantar pressure and EMG signals. Kim et al. (27), using RAS, also found significantly increased external rotation of the hip joint at initial contact which brings the CP patients closer to the external rotation range of the TD control group.

One multi-session study reported increased hip flexion at initial contact (25). Kim et al. (22) and Gagliardi et al. (39) both observed increased hip extension, with Kim et al. (22) reporting improvements during terminal stance and Gagliardi et al. (39) noting enhanced range of motion through improved minimum hip flexion. Similarly, Kim et al. (28) found reduced excessive hip flexion, though they also reported worsening of hip adduction and internal rotation. In addition, Spomer et al. (25) observed greater hip flexion during early and late swing phases, while another study found no significant changes (17).

### Pelvic kinematics

Pelvic kinematics as an important outcome measure in several studies revealed mixed results regarding intervention effectiveness. In single-session studies, Van Gelder et al. (35) found that feedback on hip angle significantly increased both pelvic tilt ROM and pelvic obliquity ROM, though this came at the cost of increased trunk and pelvis deviations from normal gait patterns. Tactile biofeedback guidance guided pelvic biomechanics towards a healthy gait pattern, though pelvic obliquity of CP participants remained significantly higher compared to the control group (30). Other single-session studies found no significant effects on pelvic parameters (20, 36).

Multi-session studies demonstrated more consistent improvements, with Kim et al. (22) showing significant increase of anterior pelvic tilt at initial contact and throughout the gait cycle after RAS intervention. Gagliardi et al. (39) using visual feedback measured improvements in both pelvic tilt ROM and pelvic obliquity ROM bilaterally. However, Spomer et al. (25) found no significant differences in pelvic kinematics, using an audiovisual biofeedback modality.

### Enhanced neuromuscular parameters

Spasticity and joint stiffness, caused by overactivation of lower limb muscles, are major contributors to gait abnormalities in individuals with cerebral palsy (CP). Therefore, many studies focused on altering muscle activation patterns using augmented feedback (AF) interventions.

### Lower leg muscles

In the lower leg the soleus and gastrocnemius, play a critical role in plantarflexing the ankle during the push-off phase, which stabilizes the body and propels the center of mass forward. All studies that measured soleus and gastrocnemius muscle activity reported positive effects of feedback interventions. In the single session studies, an increased activation of the plantar flexors and reduced co-activation with the tibialis anterior was found (19, 20). Fang and Lerner (20) observed a 9% increase in soleus activity and a 22% increase in gastrocnemius activity, while Conner et al. (19) reported 58%–82% increases in these muscles. Flux et al. (36) compared EMG-based feedback during early stance, push-off, and both time points combined and reported a decrease of 6.8% in triceps surae activity during early stance after early stance and combined feedback and increased activity of 8.1% during push-off.

In multi-session studies, results showed similar positive effects. Spomer et al. (25) reported a 28.5% increase in soleus activity during late stance when providing audiovisual feedback on soleus activity. These effects however were short-lasting once the feedback was discontinued. Other studies, such as Colborne et al. (17), reported long-term decreases in excessive triceps surae activity, but did not report information if the results were significant or if they performed a statistical analysis.

### Upper leg muscles

The vastus lateralis, responsible for knee stabilization and shock absorption during early stance, was the main upper leg muscle studied. In their single-session study Fang and Lerner (20) reported a 19.6% increase in vastus lateralis activity during incline walking. However, in multi-session studies no significant changes in upper leg muscle activation reported (17, 25).

### Ankle co-contraction index and muscle synergy adaptations

The ankle co-contraction index measures how well the muscles around the ankle work together, indicating coordination and stability. In the single-session study of Conner et al. (19), a significant 52% increase in the ankle co-contraction index was reported when using EMG-based feedback, but not with plantar-pressure-based feedback. Booth et al. (13) focused on muscle synergy changes. The changes in muscle synergies were limited to only a few significant adaptations, including the number of synergies that are required to explain at least 90% of the variance of muscle activation, but there were no significant changes in the complexity of the synergies. In the multi-session studies, Spomer et al. (25) reported no significant changes in the co-contraction index after audiovisual feedback.

**Figure 4:**
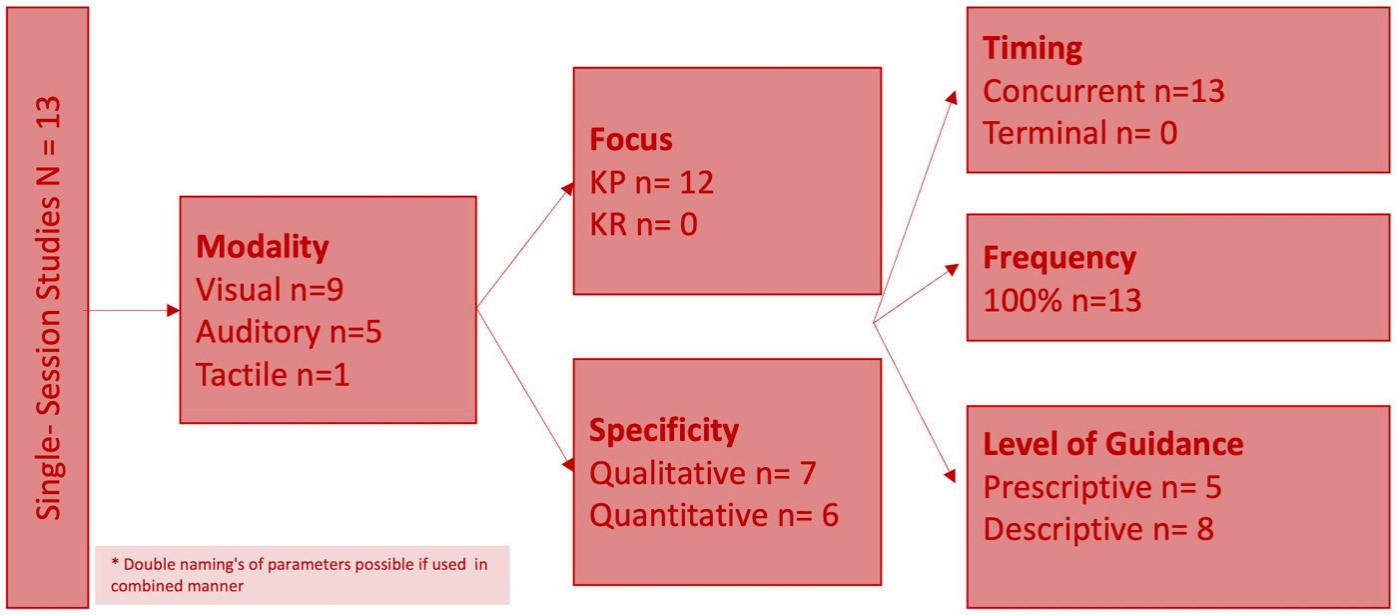
Feedback characteristics of single-session studies.

**Figure 5:**
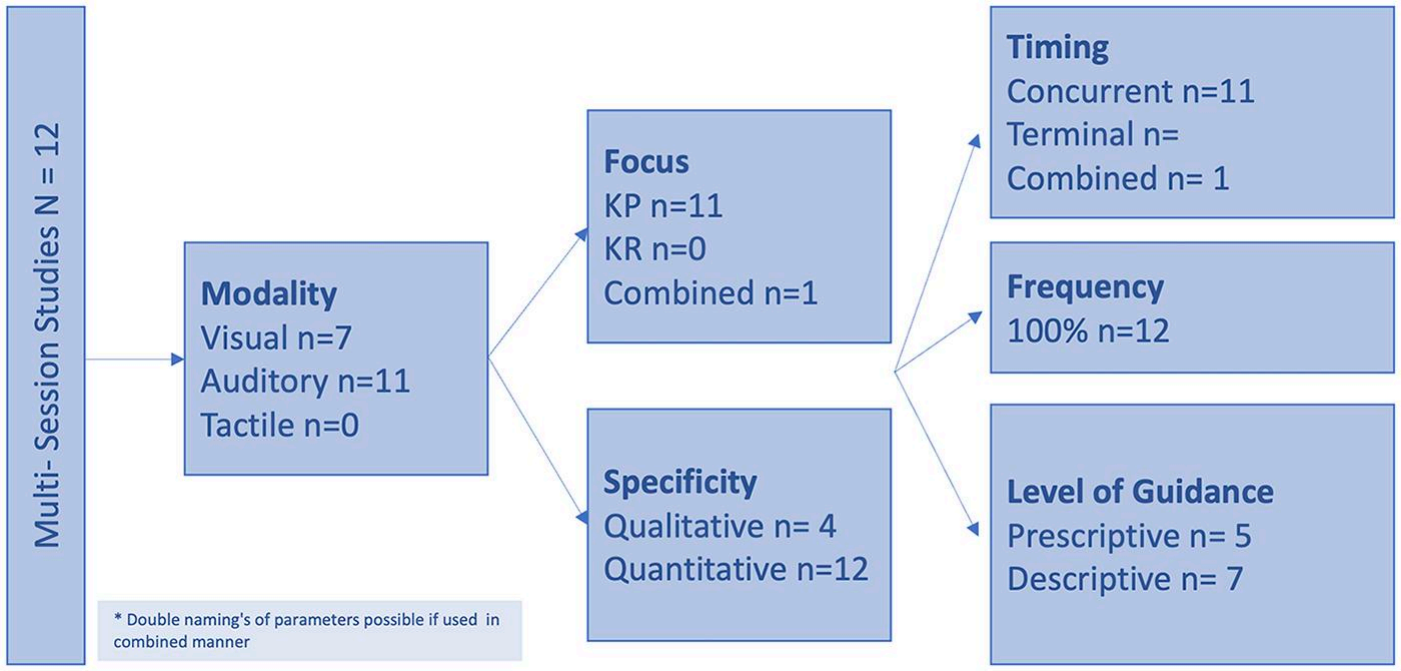
Feedback characteristics of single-session studies.

A more distinct overview about the study characteristics is shown in Figure 4 for the single-session studies and Figure 5 for the multi-session studies.

### Level of evidence synthesis

Among the spatiotemporal gait parameters, only velocity showed a strong level of evidence for improvement, supported by consistent findings across multiple high-quality studies. Similarly, increases in ankle kinematics demonstrated strong evidence, while gastrocnemius and soleus activation showed moderate evidence of enhancement. In contrast, findings related to step length, stride length, step width, cadence, knee and hip kinematics, pelvis motion, and muscle synergies were inconsistent or conflicting, resulting in inconclusive evidence. Changes in muscle synergy complexity was supported by a single high-quality study (13), leading to limited evidence. For quadriceps, tibialis anterior, and ankle co-contraction, conflicting or insufficient data also rendered the evidence inconclusive. Overall, while isolated improvements in specific parameters were observed, particularly in velocity and ankle function, the broader evidence base remains mixed, highlighting the need for further high-quality, consistent research. More detailed information can be found in Table 2.

## Discussion

### Key findings

The objective of this systematic review was to evaluate the effects of augmented feedback on gait parameters in individuals with cerebral palsy and to identify optimal feedback parameters for improving rehabilitation outcomes. This review included 25 studies (13 single-session, 12 multi-session) comprising 409 participants, representing a comprehensive analysis of the current evidence base. The studies included in the review showed considerable heterogeneity in terms of study design, gait tasks employed, outcome measures, intervention protocols, and overall study quality. This variability limits the ability to draw clear conclusions regarding the role of AF in gait rehabilitation in CP and did not allow quantitative meta-analysis to be performed due to substantial differences in outcome measurement methods, intervention protocols, feedback modalities, and participant characteristics across studies. The high degree of clinical and methodological heterogeneity, combined with inconsistent reporting of effect sizes and confidence intervals, made statistical pooling inappropriate and potentially misleading. However, the findings indicate that AF can initiate positive effects on gait performance in patients with mild spastic CP, particularly with respect to improvements in ankle joint function. Spatiotemporal parameters emerged as the most frequently assessed outcome measures, with changes in ankle kinematics being the most consistently reported improvement. Based on our level of evidence synthesis, velocity improvements showed strong evidence (consistently supported by high-quality studies), while ankle kinematics demonstrated moderate to strong evidence. However, many other parameters including step length, stride length, and cadence showed inconclusive evidence due to conflicting findings or reliance on lower-quality studies. Visual feedback modalities, particularly using concurrent and continuous knowledge of performance (KP), showed the most consistent positive effects across outcomes, though auditory feedback demonstrated specific benefits for temporal gait parameters**.** Thus the specific parameters upon which feedback was based seemed to not significantly influence the outcomes. Additionally, the duration of feedback sessions and the dosage of interventions varied significantly across studies. Long-term retention of effects appeared promising, but few studies conducted follow-up assessments, and those that did mostly employed short follow-up intervals.

Study quality assessment revealed significant variability, with eight studies rated as high quality, 13 as moderate quality, and four as low quality. This distribution directly influenced the strength of evidence for different outcomes, with higher-quality studies providing more reliable support for ankle kinematics and velocity improvements.

### Improvements of impaired gait in spastic CP

Significant improvements in gait patterns were found among patients with spastic CP across various outcome measures, indicating that AF may benefit this subgroup. However, caution is necessary when generalizing these findings to other CP types, as different brain regions may require tailored feedback strategies (1). The majority of included studies (20 out of 25) focused specifically on spastic CP, with most participants classified as GMFCS levels I and II, limiting generalizability to more severely affected individuals. Several studies used typically developing children as control groups, which may introduce bias since these children's gait patterns are already highly optimized (40). This limits the interpretation of feedback intervention results compared to CP patients. Most participants were classified as GMFCS level II, suggesting that individuals with milder impairments may respond better to AF. This raises questions about who benefits most from this approach and whether it could be counterproductive for some patients. Research shows variability in responses to AF, with some studies indicating that individuals with lower motor abilities benefit more (26, 36), while others suggest higher abilities correlate with better outcomes (20). Age also plays a key role when designing interventions, with evidence supporting better responses from younger patients (26) while others report more significant improvements for older individuals (13). Tailoring feedback to specific age groups could enhance efficacy (4). Flodmark (41) found positive responses in children with spastic hemiplegia and diplegia, but not in those with cognitive impairments, highlighting the need for further exploration of these distinctions (3).

Assessment of responder characteristics involved analyzing differences in outcome measures post-feedback, with variability likely stemming from the diverse measures used. The comparability of these measures will be addressed in the next section.

### Standardized quantification of gait parameters

The quantification of gait improvements was complicated by the use of diverse outcome parameters across the included studies, which were often adapted to specific research goals. For a more comprehensive assessment of AF effects, utilizing a standardized reliable clinical gait analysis framework that encompasses spatiotemporal, kinematic, and kinetic parameters is essential (3). Several of the included parameters measured are clinically relevant, yet the variation in measurements across studies hinders comparison.

The GMFM-88 is a validated tool for evaluating motor function and gait abilities in CP patients (42, 43) however, only two studies utilized it (17, 39). Future research should adopt standardized clinical assessments to enhance comparability, particularly using the GMFM-88, which includes dimensions D and E that evaluate standing and dynamic movements like jumping and walking (3). While GMFM-88 does not provide detailed spatiotemporal neuromuscular or kinematic gait parameters, its use reflects an effort toward standardization and comparability across studies. This highlights a broader issue in the field: many studies use diverse assessment tools, making it difficult to compare outcomes. Future research should prioritize the use of validated and standardized gait analysis methods, such as 3D motion capture, EMG analysis, or instrumented gait analysis, to improve cross-study comparability and data synthesis.

Another important aspect concerns whether improvements in gait parameters translate to enhanced quality of life for CP patients. While improved gait is a key aspect of rehabilitation (70, 71), Kerr et al. (73) found no correlation between gait energy efficiency and participation restrictions, suggesting that better gait does not necessarily increase participation in everyday life. Future studies could benefit from integrating questionnaires to assess short- and long-term impacts on quality of life.

Despite the variability in gait measures, some consistent positive effects of AF were observed, particularly in ankle kinematics, which will be further explored in the next section.

### Changes in ankle joint kinematics through augmented feedback

Changes in the ankle joint were the most consistent adaptation across studies and demonstrated one of the strongest level of evidence in our synthesis. As the most distal joint, the ankle is crucial for initiating ground contact in early stance and facilitating push-off in late stance (9). High-quality studies consistently demonstrated improvements in ankle power generation, range of motion, and plantarflexor muscle activation, particularly when EMG-based visual feedback was employed (13, 25, 36).

About half of the studies used treadmill walking, which differs significantly from ground walking. Research shows that children with CP exhibit distinct kinetic outcomes on a treadmill, including shifts from ankle to hip strategies, increased ankle power during early stance, reduced step length, and wider step width (44, 45). Additionally, joint angles vary, with greater knee flexion observed at initial contact (44). Thus, findings from treadmill protocols should be interpreted cautiously, as they may not apply directly to overground walking, limiting external validity. No studies have yet examined the transferability of AF intervention effects from treadmill to overground conditions, indicating a gap for future research.

Several studies reported an increased RoM in the ankle and knee joint. This increase may suggest reduced joint stiffness, a major factor in gait abnormalities in spastic CP (10, 46). Abnormally high stiffness may be attributed to neural factors like spasticity or non-neural factors linked to altered tissue properties (10). Effective gait rehabilitation must address the underlying causes of stiffness. AF as an intervention focuses on changing muscle activity which may then also affect tissue properties in the long term. Indeed, conditioning of abnormally high reflexes in spastic spinal cord patients has shown a significant potential in reducing spasticity without the need for medications (47, 48). However, projecting these findings to CP populations requires caution, as the underlying pathophysiology differs significantly from spinal cord injury. While AF may contribute to improved motor control and potentially influence tissue properties over time, claims about reducing surgical interventions in CP remain speculative and require further investigation. Thus, individualized AF designs, based on thorough clinical evaluations, is essential for targeting common gait limitations.

### The role of feedback modality and the parameter the feedback is based on in improved gait

The studies included in this review utilized a variety of feedback modalities, ranging from visual, auditory, and audiovisual. However, none incorporated tactile feedback, such as vibration or pressure except for one recent study (30) that demonstrated promising results with tactile vibration feedback for trunk control**.** Based on our evidence synthesis, visual feedback showed the strongest and most consistent effects across multiple gait parameters, particularly when delivered as concurrent, continuous knowledge of performance (KP). Auditory feedback, especially rhythmic auditory stimulation (RAS), demonstrated specific benefits for temporal parameters like cadence and stride timing.

An important distinction between general auditory feedback and rhythmic auditory stimulation (RAS) has to be pointed out. While both fall under the auditory modality, RAS specifically targets temporal gait aspects through rhythmic cueing, whereas general auditory feedback may provide information about various gait and performance parameters without the rhythmic aspect. Studies using RAS consistently showed improvements in temporal parameters, with Kim et al. (28) suggesting that RAS primarily affects pelvic and hip movement rather than distal movements of the knee, ankle, or foot.

**Figure 6:**
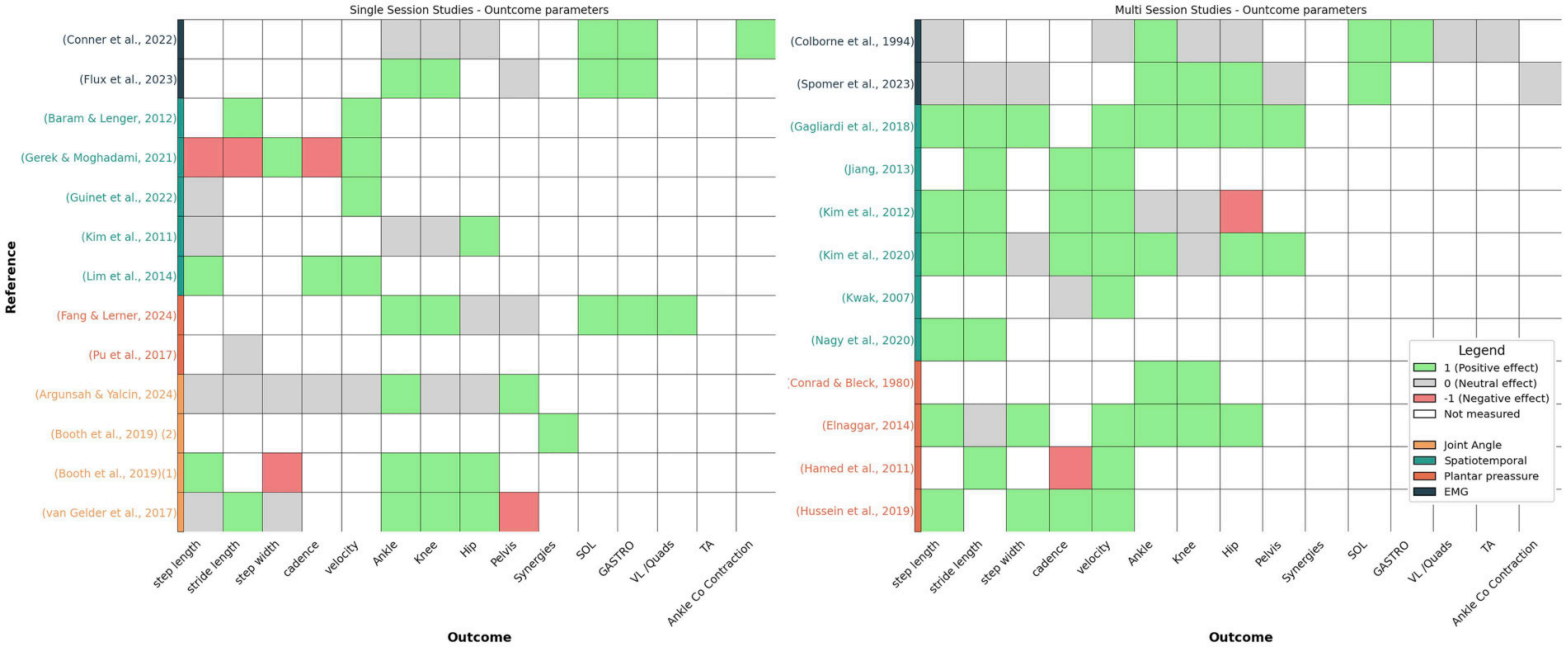
Single-session studies (Left) and multi-session studies (Right) in relation to the parameter the feedback was based on (colour of the reference) and the significant outcomes that increased (green), decreased (red), did not show significant effects (grey), and were not measured (white).

Certain feedback modalities were frequently paired with specific parameters as shown in Figure 6. Auditory feedback was typically used for plantar pressure and spatiotemporal data, while visual and audiovisual feedback were more commonly applied to kinematic and EMG data. This correlation may give the misleading impression that only certain modalities are effective for specific outcomes. However, the modality and measured outcomes were often aligned, as studies providing feedback on kinematic data primarily assessed kinematic outcomes, and similarly for EMG.

Another notable trend was the increased use of visual and audiovisual feedback in more recent studies, likely due to advancements in data visualization technologies (13). However, approximately 34% of individuals with CP, especially younger children, experience cerebral visual impairment, making visual feedback less suitable for some patients (49). For these individuals, auditory feedback may be more effective. Selecting feedback modalities and parameters should be tailored to the individual's needs, especially considering sensory impairments (1).

The differential effectiveness of feedback modalities can be attributed to several neurophysiological and practical factors. Visual feedback appears particularly effective for kinematic parameters because it may leverage the brain's robust visual-motor integration pathways, allowing patients to directly observe and correct movement patterns in real-time (50). This is especially beneficial for individuals with spastic CP who retain intact visual processing, as visual feedback may bypass impaired proprioceptive pathways and provide clear, interpretable movement information (51). However, the approximately 35%–85% prevalence (52, 53) of cerebral visual impairment in CP populations limits visual feedback applicability. Particularly in individuals with higher GMFCS levels, where the percentage of visual impairments increases as well (53).

Auditory feedback, especially RAS, demonstrates superior effectiveness for spatiotemporal parameters due to the strong neural coupling between auditory processing and motor timing networks in the brainstem and cerebellum (8, 28). The rhythmic nature of auditory cues can entrain motor patterns through subcortical pathways that may remain relatively preserved in CP (29). Additionally, auditory feedback offers advantages in patients with visual impairments and may be less cognitively demanding than complex visual displays. The often lower technological requirements of auditory feedback systems also enhance clinical feasibility compared to sophisticated visual display systems (8).

### Implications for feedback scheduling, timing, and long-term effects

#### Session duration and frequency

The duration of feedback sessions varied significantly across studies, making it difficult to draw firm conclusions. Nearly half of the studies (13 out of 25) were single-session studies. As shown in several studies with stroke patients, a single session gait training protocol typically results in immediate effects on gait parameters (54, 55) which may not be sustained in the long term (55).

In multi-session studies, session length and frequency also varied, a trend seen in other reviews on augmented feedback in CP (5, 6). State-of-the-art gait rehabilitation methods, like robotic or body weight-assisted training, usually last 30 to 60 min, 2–5 times per week (71), but it remains unclear if this also applies to augmented feedback interventions.

The studies included participants from various age groups, with many focusing on children but also representing adults. While developing a near-normal gait pattern early in life is crucial, research shows that gait abilities tend to decline with age in adults with CP (74), highlighting the need for targeted gait interventions across all ages. Motor learning evolves significantly during childhood (75), and declines in motor learning capacity have been observed in older adults (77). Cognitive impairments can further impact motor learning (76), making it important to tailor the duration and frequency of feedback to suit both age and cognitive abilities (4).

All studies used continuous feedback, but when considering that constant feedback may lead to cognitive overload and dependency on the feedback system, this may limit the patients' use of intrinsic feedback like proprioception (4, 56). For complex motor tasks such as walking, high feedback availability therefore may help in early learning phases (57) but a “faded” feedback approach—intense at first, then gradually reduced—may be more beneficial to promote long-term skill transfer.

#### Long-term effects

Evaluating long-term effects is a key challenge in gait rehabilitation to truly improve patient participation in activities of daily living (58). Only five studies conducted long-term assessments with very small sample sizes, limiting the ability to draw conclusions about the lasting effects of AF. This was also mentioned in other reviews of AF in CP (5, 6) and is also a major limitation of this review. The limited follow-up data represents a critical gap in the evidence base, as immediate improvements do not necessarily translate to functional improvements in daily activities or long-term motor learning benefits.

### Level of evidence synthesis and study quality implications

Our systematic evidence synthesis revealed variability in the strength of evidence across different gait parameters (Table 3). We found that velocity improvements demonstrated the strongest evidence level, being consistently supported by multiple high-quality studies (22, 28, 39). Ankle kinematics showed moderate to strong evidence, particularly for ankle power generation and range of motion, supported by several high-quality studies employing EMG-based feedback systems.

**Table 3:** Level of evidence synthesis for individual outcome parameters.

Outcome parameter	Effect direction	Association (studies)	No association/opposite	Level of evidence
Step Length	Increase	(13, 22, 23, 28, 31, 33, 37, 39)	(17, 21, 25, 27, 30, 34, 35)	Inconclusive
Stride Length	Increase	(22, 26, 28, 32, 33, 35, 38, 39)	(21, 24, 25, 30, 31)	Inconclusive
Step Width	Decrease	(21, 31, 37, 39)	(13, 22, 25, 35)	Inconclusive
Cadence	Increase	(22, 23, 28, 37, 38)	(21, 29, 30, 32)	Inconclusive
Velocity	Increase	(21–23, 28, 29, 31, 32, 34, 37–39)	(17, 30)	Strong
Ankle	Increase	(13, 17, 18, 20, 22, 25, 30, 31, 35, 36, 39)	(19, 27, 28)	Strong
Knee	Increase	(13, 18, 20, 25, 31, 35, 36, 39)	(17, 19, 22, 27, 28, 30)	Inconclusive
Hip	Increase	(14, 22, 25, 27, 31, 35, 39)	(17, 19, 20, 28, 30)	Inconclusive
Pelvis	Decrease	(22, 30, 39)	(20, 25, 35, 36)	Inconclusive
Muscle Synergies	Increase	(14)	–	Limited
Soleus (SOL) Activation	Increase	(17, 19, 20, 25, 36)	–	Moderate
Gastrocnemius (GASTRO) Activation	Increase	(17, 19, 20, 36)	–	Moderate
Quadriceps (VL/Quad)	Increase	(20)	(17)	Inconclusive
Tibialis Anterior (TA)	Increase	–	(17)	Inconclusive
Ankle Co-Contraction	Decrease	(19)	(25)	Inconclusive

In contrast, many spatiotemporal parameters including step length, stride length, and cadence showed inconclusive evidence due to conflicting findings across studies of varying quality. The heterogeneity in study quality significantly influenced these results, with lower-quality studies often reporting inconsistent or contradictory findings. This pattern underscores the importance of rigorous study design and standardized outcome measures in AF research.

The distribution of study quality (32% high, 52% moderate, 16% low) highlights the need for more high-quality research to strengthen the evidence base. Studies rated as low quality were primarily limited by small sample sizes, lack of control groups or insufficient reporting of significant results, factors that directly impact the reliability of their findings.

### Impact of study biases on conclusions

The identified biases in included studies significantly influence the interpretation of our findings and limit the generalizability of conclusions. The predominant use of typically developing children as control groups (seen in 40% of studies) introduces a fundamental bias, as these children possess optimized gait patterns that may not reflect realistic improvement targets for CP patients. This bias may amplify effect sizes and creates unrealistic expectations for therapeutic outcomes, as the comparison represents an idealized rather than clinically relevant benchmark.

Sample size limitations affected 60% of studies, with many reporting fewer than 15 participants per group. This underpowering substantially increases the risk of both Type I and Type II errors, potentially leading to false-positive findings for some parameters while missing genuine but smaller effects for others. The predominant focus on GMFCS levels I and II (representing 85% of all participants) creates a selection bias that limits applicability to more severely affected individuals, who may actually benefit most from augmented feedback interventions but require different implementation strategies.

The heterogeneity in outcome measures further adds to these biases, as studies may inadvertently select measures that favor their specific intervention approach, creating an appearance of effectiveness that may not translate to standardized clinical assessments.

### Limitations

This systematic review has several limitations that need to be addressed. First, the decision to include various study designs (RCTs, crossover studies, single-group pre-post designs) was made to capture the full scope of available evidence in this emerging field, recognizing that while RCTs provide the strongest evidence, the limited number of high-quality RCTs would have significantly restricted our ability to comprehensively evaluate AF effects. This inclusive approach, while potentially limiting the strength of conclusions, provides a more complete picture of the current evidence landscape and identifies areas where higher-quality research is urgently needed. To overcome this limitation to some degree, we performed a level of evidence assessement. Additionally, it was not possible to compare AF directly to state-of-the-art gait rehabilitation approaches such as robotic-assisted training or functional electrical stimulation, as included studies primarily used traditional gait training or no-feedback controls.

Second, interventions involving robotic or exoskeleton-based feedback were excluded because these systems provide externally-generated tactile and mechanical feedback rather than self-produced feedback. This distinction was important to maintain focus on interventions that enhance the patient's own sensory feedback systems rather than replacing them with external mechanical assistance. However, this exclusion may have limited our understanding of the broader landscape of feedback-enhanced gait rehabilitation.

The main limitation was the lack of statistical comparison between the outcomes of the studies, which is essential to quantify the significance of findings. This was mainly due to the heterogeneity of outcome measures, as most studies used different methods, evaluation tests or gait parameters. Therefore, this systematic review summarizes the main findings in a descriptive manner, albeit providing a level of evidence synthesis and discusses the implications of AF for gait rehabilitation in CP. This descriptive approach, while necessary given the heterogeneity, limits our ability to provide definitive clinical recommendations and effect size estimates. Importantly this highlights the urgent need for more stringent RCT study designs which are both complex and costly.

## Conclusion and outlook for future research

This review aimed to evaluate the effects of AF in gait rehabilitation for CP. Overall, AF showed promising results in improving gait parameters, particularly in spastic CP. However, the evidence remains limited, and it is difficult to make definitive conclusions for all CP subtypes. The studies reviewed demonstrated that AF has a greater impact on gait function than traditional therapy alone, benefiting both kinematic and neuromuscular aspects. Despite these positive trends, the heterogeneity of the studies made it challenging to draw comprehensive conclusions.

Our level of evidence synthesis revealed that while some parameters like velocity and ankle kinematics show strong to moderate evidence for improvement, many other gait parameters remain inconclusive due to study quality limitations and conflicting findings (Table 3). Importantly, the methodological quality of the included studies varied considerably, which affects the overall confidence in the reported effects. While some parameters such as velocity and ankle kinematics were supported by consistent findings from high-quality studies (indicating strong evidence), many other outcomes showed inconclusive or limited evidence due to inconsistent findings or reliance on lower-quality data. Therefore, the interpretation of the benefits of AF should be made with caution, especially in areas where study quality was low or results were conflicting.

Key areas for future research include assessing the long-term effects and transferability of AF, establishing guidelines for its duration and dosage, and determining the most effective feedback designs for specific gait abnormalities. Future studies should prioritize randomized controlled trial designs with adequate sample sizes, standardized outcome measures, and longer follow-up periods to address the current evidence gaps. Developing easy-to-use devices for home-based AF practice and investigating potential long-term effects, such as feedback dependency, are also important. Additionally, protocols from single-session studies should be tested in multi-session interventions to evaluate long-term outcomes.

In summary, AF appears to be a promising tool for gait rehabilitation in children with CP, particularly for improving velocity and ankle kinematics. However, current findings are constrained by methodological limitations, such as small sample sizes, inconsistent outcome measures, and participant selection biases. These factors limit the strength and generalizability of the conclusions. While early results are encouraging, especially in spastic CP, the evidence remains insufficient to support widespread clinical adoption. Well-designed, large-scale studies are essential to validate the effectiveness of AF, explore its long-term impact, and inform evidence-based guidelines for clinical implementation.
