# Supplementary material for: Augmented feedback as a therapeutic approach for gait rehabilitation in patients with cerebral palsy: a systematic review
Source: Front Rehabil Sci. 2026 Mar 11;7:1638091. doi: 10.3389/fresc.2026.1638091 (PMC13013304; doi:10.3389/fresc.2026.1638091)
Supplement: Supplementary file 1 [file Datasheet1.pdf]

Supplementary Material

Additional search terms for included Libraries:

Cochrane library

(cerebral palsy OR CP) AND (((augmented OR extrinsic OR external OR verbal OR visual OR video OR auditory OR haptic OR tactile OR sensory OR robot* OR multi-modal OR multimodal OR bio OR neuro OR vibrotactile) AND feedback) OR neurofeedback OR biofeedback OR visual augmented feedback OR audio augmented feedback OR multi-modal augmented feedback OR knowledge of performance OR knowledge of result* OR enhanced feedback OR feedback strateg*) AND (walk* speed OR gait speed OR gait velocity OR walk velocity OR Step length OR Gait width OR mobility test OR spatiotemporal OR walk* test OR Up and go OR Berg Balance Scale OR Physiological cost index OR Walking handicap scale OR (Functional Ambulation AND (Index OR Score)) OR step frequency OR cadence)

PEDro Library

Cerebral palsy AND feedback

Cerebral palsy AND neurofeedback

cerebral palsy AND biofeedback

cerebral palsy AND knowledge of performance

cerebral palsy AND knowledge of result

IEEE Xplore

(cerebral palsy OR CP) AND (((augmented OR extrinsic OR external OR verbal OR visual OR video OR auditory OR haptic OR tactile OR sensory OR robot* OR multi-modal OR multimodal OR bio OR neuro OR vibrotactile) AND feedback) OR neurofeedback OR biofeedback OR visual augmented feedback OR audio augmented feedback OR multi-modal augmented feedback OR knowledge of performance OR knowledge of result* OR enhanced feedback OR feedback strateg*) AND (walk* speed OR gait speed OR gait velocity OR walk velocity OR Step length OR Gait width OR mobility test OR spatiotemporal OR walk* test OR Up and go OR Berg Balance Scale OR Physiological cost index OR Walking handicap scale OR (Functional Ambulation AND (Index OR Score)) OR step frequency OR cadence)
